# Lectotypifications, synonymy, and a new name in *Capsicum* (Solanoideae, Solanaceae)

**DOI:** 10.3897/PhytoKeys.2.730

**Published:** 2011-02-11

**Authors:** Gloria E. Barboza

**Affiliations:** Instituto Multidisciplinario de Biología Vegetal (IMBIV-CONICET), and Pharmacy Departament, Chemical Science Faculty, National University of Córdoba, Casilla de Correo 495, 5000 Córdoba, Argentina

**Keywords:** *Capsicum*, nomenclature, taxonomy, typification, “ulupicas”

## Abstract

Considerable confusion exists within Capsicum (Solanaceae) regarding the status and typification of several names, in part due to misidentifications. Some types were destroyed in Berlin during the Second World War, some have not been found by modern systematics, while others exhibit uncertain locality data or contain material from more than one species. Fourteen lectotypes, synonyms, and a new name, Capsicum eshbaughii Barboza **nom. nov.**,are proposed here.

## Introduction

Capsicum L. (Solanaceae) comprises approximately 32 species, including five species (Capsicum annuum L., Capsicum frutescens L., Capsicum chinense Jacq., Capsicum baccatum L., and Capsicum pubescens Ruiz & Pav.) known for their pungent fruits under the common names “chile”, “ají”, “paprika”, “chili”, “chilli pepper”, “tabasco”, “habanero”, pimenta-de-cheiro”, “rocoto”, etc. and the non-pungent Capsicum annuum cultivars known as “sweet bell pepper” and “pimiento”.

This genus has been known since the beginning of civilizations in the Western Hemisphere and has been part of the human diet since 6000–7500 BC (Basu and De 2003; Perry et al. 2007). After Columbus introduced Capsicum seeds into Spain, the crop was widely spread all over the world. Currently, five domesticated species are cultivated in many countries for their great economic value as vegetables, pungent food additives, colourants, pharmaceuticals, and even as popular medicines (Bosland and Votava 2000; Reifschneider 2000; Buckenhüskes 2003; Thampi 2003; Ravishankar et al. 2003; Barceloux 2008; Yamamoto and Nawata 2009). The fruits of most Capsicum species contain significant quantities of a great variety of metabolites (vitamins, carotenoids, minerals, proteins, carbohydrates, fats, fibre) but their importance is derived from their possession of the major pungent compounds capsaicin and dihydrocapsaicin (Bosland and Zewdie 2001; Pruthi 2003; Manirakiza et al. 2003) which accumulate in the secretory epidermis of the septum (Filippa and Bernardello 1992).

Tournefort (1719: 152) gave the name Capsicum to the genus which was later taken up by Linnaeus (1753: 188); since then there has been no consensus about the number of species included in it or its circumscription. Many species recognized as Capsicum today were originally described in different solanaceous genera such as Acnistus Schott, Bassovia Aubl., Brachistus Miers, Fregirardia Dunal ex Delile, Solanum L., and Witheringia L’Hér., while species now placed in diverse genera were originally described as members of Capsicum. The delimitation of Capsicum was chaotic until Hunziker and collaborators provided revisions of the morphologically similar genera Witheringia (Hunziker 1969), Acnistus (Hunziker 1982), Vassobia Rusby (Hunziker 1977, 1984), Athenaea Sendtn. (Barboza and Hunziker 1989), Aureliana Sendtn. (Hunziker and Barboza 1991), and Lycianthes (Dunal) Hassl. (Barboza and Hunziker 1992). A convincing circumscription of Capsicum (Hunziker 2001; Barboza and Bianchetti 2005; Barboza et al. 2010) has now been made possible using flower and fruit characters.

There is currently no consensus classification of Capsicum itself. The infrageneric taxa proposed by Kuntze (1891), Wettstein (1891), Bitter (1921) and Hunziker (1956) have later been recognized as the segregate genera: Witheringia, Brachistus, Saracha Ruiz & Pav., Tubocapsicum (Wettst.) Makino, Aureliana (Hunziker 2001). More recently, different classical and molecular cytogenetic analyses, crossing experiments, enzymatic studies, and chloroplast and nuclear DNA sequence studies (see references in Moscone et al. 2007 and Barboza et al. 2010), have allowed considerable progress in the characterization of infrageneric groups in Capsicum. At present, there is no formal infrageneric classification. Two attempts at grouping species were made based on cytogenetic studies (Moscone et al. 2007), and a combination of data from enzyme, crossing and molecular studies (Walsh and Hoot 2001). In both studies the informal classification is still considered provisional despite more than 50% of the species having been analyzed.

Working towards a complete treatment of wild Capsicum species I became aware of several instances of confusion regarding erroneous or uncertain names. In addition, some type specimens have not been found or have been destroyed in Berlin during the Second World War while others exhibit uncertain locality data or are composed of material from more than one species.

In this paper, lectotypes are designated for 14 names, and these are synonymized under their accepted names in Capsicum. In addition, a new name in Capsicum is proposed. In each case below, the locality information given for the lectotype corresponds with the information found on the specimen itself.

## Lectotypifications

Acnistus geminifolius Damm., Bot. Jahrb. 36(4): 384. 1905. Type citation: [ECUADOR]. “Crescit in declivibus montis Carazou pr. Miligally (S[odiro]. n. 114/82 – Mai 1882); in silvis subandinis et subtropicis pr. Couzauho (S[odiro]. n. 114/81 – Mai 1882; in silvis m. Carazou (S[odiro]. n. 114/84)”.- Lectotype (designated here): [ECUADOR. Pichincha]. “In silv. Monte Corazón, Sep 1873, Sodiro 114/84” – P! (P00410128) = Capsicum geminifolium (Damm.) Hunz. (Hunziker 1956).

The original collections of Sodiro are thought to be deposited at QPLS and Q, both in Ecuador. No syntype collections of Acnistus geminifolium I found in these or in B, G, MO, NY, SI, or US. A photograph of the destroyed specimen at B of Sodiro 114/82 exists at F (Field Museum Negative #2487). The only collection found, Sodiro 114/84, is housed at P and is here chosen as lectotype.

Brachistus coccineus Rusby, Bull. New York Bot. Gard. 8(28): 117. 1912 ≡ Lycianthes coccinea (Rusby) Rusby, Bull. Torrey Bot. Club 53: 210. 1926. Type citation. [BOLIVIA]. “Six feet high; San Buena Ventura, 1400 ft., Nov. 30, 1901 ([Williams] Nos. 623 and 634)”.- Lectotype (designated here): [BOLIVIA. Dpto. La Paz, Prov. A. Iturralde]. “San Buena Ventura, 1400 ft, 8 Nov 1901, R.S.Williams 634”.- NY! (NY00138552); isolectotypes: BM! (BM0000884131), K! = Capsicum coccineum (Rusby) Hunz. (Hunziker 1956).

Williams 634 at NY is the best and the most complete of the two cited Williams collections, with flower buds, flowers and mature fruits; it is selected here as lectotype. The second collection (Williams 623: BM!, K!, NY!, US!) is predominantly in fruit. After describing Brachistus coccineus, Rusby (1926) transferred his species to Lycianthes, a placement later accepted by Morton (1944). This was due to Rusby’s inspiration by Bitter´s monograph on Lycianthes (Bitter 1919), which states that the possession of calyx teeth is a basic feature of Lycianthes. Hunziker (1956), on the other hand, emphasized the importance of the androecium (more so than the calyx) for the generic delimitation of Capsicum and retained Capsicum coccineum. In relation to Brachistus coccineus, Hunziker noted that the anthers are longitudinally dehiscent and the typical prominent stapet (base of the filament broadened and fused to the corolla tube, with lateral auriculate appendages) of Capsicum species present in both syntypes.

Brachistus hookerianus Miers, Ann. Mag. Nat. Hist., ser. 2, 3 (16): 268. 1849. Type citation: “Ecuador, v. s. in herb. Hook. (Cerro de Lantana, Guayaquil, Jameson, et in horto Kewensis cultus)”. – Lectotype (designated here): [ECUADOR]. “Guayaquil, Cerro of Lantana, Jan 1846, W.Jameson s.n.” – K! (K000585919); isolectotype: US! photo + fragm. = Capsicum hookerianum (Miers) Kuntze (Jørgensen and León-Yánez 1999).

Analysis of the original material suggests that Miers (1849) described Brachistus hookerianus based mostly on the plant cultivated at Kew Gardens, now representated as a specimen at K. This specimen (with a duplicate at BM) has a small pubescent calyx with entire margin and 5 teeth, and a subcampanulate corolla as described in Miers’ (1849) protologue. Three sheets of Jameson’s field collection from Ecuador are deposited in Kew as Brachistus hookerianus. In two of them (K000585918!, K000585920!), the only label data present is “Guayaquil” whereas in the third sheet (K000585919!), the label information is exactly as in the protologue. In addition, this specimen and a fragment of it at US show a 10-toothed calyx and a subcampanulate corolla. My analysis of the cultivated specimens at K and BM cited by Miers indicates that they belong to Capsicum rhomboideum (Dunal) Kuntze. Hence the original material of Brachistus hookerianus belongs to more than one taxon. Jameson´s field collected material matches current usage of the name Capsicum hookerianum (calyx conspicuously 10-toothed, corolla brilliant yellow, subcampanulate to campanulate, mostly with simple hairs, and leaves strongly attenuate) and is selected here as the lectotype.

Brachistus pubescens Stewart, Proc. Calif. Acad. Sci., ser. 4, 1: 137. 1911 **≡** Capsicum galapagense Heiser & P.G.Sm., Brittonia 10: 200. 1958. Type citation. [ECUADOR]. “Albemarle Isl.: Villamil, bushes in woodland, 450–600 ft. ([Stewart] nos. 3351–3352). James Isl.: James Bay, occasional bushes above 1600 ft. ([Stewart] nº. 3353)”.- Lectotype (designated here): [ECUADOR. Galapagos: Isla Isabela]. “Albemarle Island, Villamil, 450–600 ft., bushes in woodland, 3 Jan 1906, A.Stewart 3352”. CAS!; isolectotypes: GH!, US! = Capsicum galapagoense Hunz. (Hunziker 1956).

The transfer of Brachistus pubescens to Capsicum necessitates a new epithet due to the earlier publication of Capsicum pubescens Ruiz & Pav. (Ruiz and Pavón 1799). Both Hunziker (1956) and Heiser and Smith (1958) chose epithets alluding to its origin in the Galapagos Islands, with Hunziker´s name having priority. Erroneous type citations in Jørgensen and León-Yánez(1999) citing Stewart 3353 at CAS as the holotype, with an isotype at GH should be corrected to syntype and isosyntype respectively (see ICBN Art. 9.8; McNeill et al. 2006). Only two of the syntype collections have been critically examined (Stewart 3351: CAS!, GH!, NY!, MO!, US!; Stewart 3352: CAS!, GH!, US!); no duplicates of Stewart 3353 could be found at either CAS nor GH. The best preserved of these specimens is Stewart 3352 (CAS!) which includes a flower, numerous flower buds and fruits, and has label data in accordance with the protologue description; the other sheets of Stewart 3352 (GH!, US!) have mainly only flower buds. Capsicum galapagoense is rare and is the only endemic species of Capsicum in the Galapagos (Isla Isabela [Albemarle] and Santa Cruz [Indefatigable]), known as “Galápagos pepper” (McMullen 1999). It is superficially similar to Capsicum annuum var. *glabriusculum*.

Capsicum campylopodium forma *magis-puberula* Chodat, Bull. Herb. Boissier ser. 2, 2: 815. 1902, syn. nov.Type citation. [PARAGUAY]. “In silvis pr. Sapucay, Dec., [Hassler]1607; in silva Ipé-hu, Sierra Maracayu, Oct., [Hassler] 5134”.- Lectotype (designated here): [PARAGUAY]. “In altoplanitie et decliviis “Serra de Maracayú”, in silva Ipé-hu, Oct., Hassler 5134. Frutex 1–2, petala alba”- G!; isolectotypes: A!, K!, P! (P00410080, P00410081), S!, UC! (UC-944853), W! = Capsicum flexuosum Sendtn.

Chodat (1902) described this form using specimens from Paraguay he considered to belong to Capsicum campylopodium, a Brazilian endemic. The type collections mentioned in the protologue (Hassler 1607 & 5134: A!, BM!, G!, K!, P!, S!, UC!, W!), and distributed in many herbaria constitute abundant and complete material, with buds, flowers and fruits corresponding to the original description. The specimen Hassler 5134 in G is designated lectotype since it bears more flowers. These plants have non-geniculate pedicels, similar to the situation observed in Capsicum flexuosum, but not in Capsicum campylopodium (Hunziker 1998; Barboza and Bianchetti 2005). The more abundant pubescence attributed to this form in the protologue is included within the variation of Capsicum flexuosum.

Capsicum hispidum Dunal var. *glabriusculum* Dunal, Prodr. 13(1): 420. 1852. Type citation. “In Mexico circa Bejar (Berland[ier] n. 1863, in h. Moric.); circa Tampica de Tamaulipas. (Berl[andier], n. 95, in herb. Moric.)”.- Lectotype (designated here): [UNITED STATES OF AMERICA, Texas, Bexar Co. San Antonio]. “Bejar, Sep 1828, Berlandier n. 1863” P! (P00410138); isolectotypes: BM! (BM000775839), F!, G!, NY! (NY00138591), P! (P00409852) = Capsicum annuum var. *glabriusculum* (Dunal) Heiser & Pickersgill (Heiser and Pickersgill 1975).

Among the original material cited by Dunal (1852), only the first widely distributed collection was successfully located. Dunal’s protologue gives the collection locality as “In Mexico circa Bejar,” now part of Texas (USA). After many searches for the correct name for the spontaneous variety of Capsicum annuum (Shinners 1956; Heiser 1964; Heiser and Pickersgill 1969; D’Arcy and Eshbaugh 1973, 1974), Heiser and Pickersgill (1975) confirmed that Capsicum hispidum var. *glabriusculum* is the correct basionym for the wild variety of Capsicum annuum widely distributed in the Americas.

Capsicum microcarpum DC. var. *tomentosum* Chodat & Hassl., Bull. Herb. Boissier 2,4:80.1903. Type citation. [PARAGUAY]. “In dumetis collis Cerro hu, pr. Paraguary, Dec., [Hassler] n. 6498, in rupestribus pr. Cerro pyta, Febr., [Hassler] n. 1926”.- Lectotype (designated here): [PARAGUAY]. “Prope Paraguarí, in dumetis collis Cerro Hu, Dec 1900, E.Hassler 6498. Suffrutex 1–1.5 petala albovirentia interne avellanes punctata” – G!; isolectotypes: BM! (BM000087632a), K! (K000585894), P! (P00410166), MO!, S!, UC!, W! = Capsicum baccatum L. var.*baccatum* (Hunziker 1998).

Chodat and Hassler (1903) described this variety based on two very pubescent collections. Both specimens are complete and match the diagnosis, but the second one (Hassler 1926: BM!, G!, P!) has been found in fewer herbaria. Thus, I designate the widely distributed collection Hassler 6498 held in G as the lectotype. This variety was described as a xerophytic entity with a dense indumentum. Pubescence is a highly variable character in many Capsicum species (Capsicum chacoense Hunz., Capsicum rhomboideum (Dunal) Kuntze, Capsicum parvifolium Sendtn., Capsicum baccatum L., among others). After studying the original material of Capsicum microcarpum var. *tomentosum*, Hunziker (1998) concluded that this taxon should be included in the range of variation existing in wild Capsicum baccatum populations. This latter species is one of the few in Capsicum with a wide range of distribution in different habitats where ecological conditions do not determine the degree of pubescence of the populations (Barboza, pers. obs.). For this reason, I agree with Hunziker’s statement since the dense pubescence of Capsicum microcarpum var. *tomentosum* does not justify the validity of this taxon but fits very well under the wild Capsicum baccatum var. *baccatum*

Capsicum microcarpum DC. var. *glabrescens* Hassl., Repert. Spec. Nov. Regni Veg. 15: 244. 1918. syn. nov. Type citation. [PARAGUAY]. “Hassler 215, 5703, 6070, 12385”.- Lectotype (designated here) [PARAGUAY]. “Iter ad Yerbales montium Sierra de Maracayu, in regione cursus superioris fluminis Jejui guazú, Dec., Hassler 5703”- G!; isolectotypes: BM!, GH!, K! (K000585896), MO! (MO503802), NY! (NY00138600), P! (P00410160, P00410161, P00482076), UC!, W! = Capsicum baccatum L. var. *baccatum*

The four syntypes of Capsicum microcarpum var. *glabrescens* have been examined. All of them are good quality complete collections preserved in many herbaria (Hassler 215: G!, K!; Hassler 6070: G!, GH!, K!, MO!, NY!, P!, W!, UC!, US!; Hassler 12385: G!, GH!, K!, L!, MO!, NY! two sheets, UC!, US!, Z!). The sheet of Hassler 5703 at G is the most complete and is designated here as lectotype. As stated above under Capsicum microcarpum var. *tomentosum*, both the tomentose and glabrescent populations of Capsicum microcarpum correspond to the entity recognized as Capsicum baccatum var. *baccatum*.

Capsicum microphyllum Dunal, Prodr. 13(1): 421. 1852, syn. nov. Type citation. “In Habanâ (de la Sagra in h. DC.), in Texas, circa Rio de Medina prope Bejar (Berland[ier] 1907, in h. Moric.)”.- Lectotype (designated here): [UNITED STATES]. “Texas, à Rio de Medina, Berlandier 1907” , G!; isolectotypes: BM! (BM000775841), GH!, MO!, NY! (NY00138590), P! (P00409956, P00409851) = Capsicum annuum var. *glabriusculum* (Dunal) Heiser & Pickersgill

Both syntypes are good quality specimens in agreement with the diagnosis. Berlandier 1907 at G is selected as the lectotype as its duplicates are broadly distributed. De la Sagra’s specimen (nº 3, [año]1888) appears to be preserved only at G-DC! Berlandier´s collections held at G!, GH! (date on label: Oct. 1828), NY!, and P! are mainly in fruit whereas the ones at BM! and MO! also have flower buds or flowers. The calyx shape and the fruit colour and shape are among the characters most useful for establishing the correct placement of this name.Thus, the small, cup-shaped and sub-dentate calyx and the globose-ovate red berry together with the presence of the solitary pedicels and white corolla (in sched. “corolla albida”) clearly observed in these specimens are good matches for Capsicum annuum var. *glabriusculum*. The small size of the leaves is within the range of variation of this wild variety.

Solanum mendax Van Heurck & Müll. Arg., Observ. Bot. 61. 1870 ≡ Capsicum mendax (Van Heurck & Müll. Arg.) J.F.Macbr., Candollea 5: 402. 1934. Type citation. “In Andibus Peruviae ecuadorensis (R.Spruce n. 5117! in hb. van Heurck et n. 5050! in hb. DC)”. Lectotype (designated here): [ECUADOR]. “Baños, in sepibus, muris delapsis Aug 1857, Spruce 5050. Frutex valde ramosus; 8-pedalis. Flores flavi. Baccae nigrae” – K! (K000201915); isolectotypes: BM! (BM000777290), C!, G!, GH!, K! (K000201792), MO! (MO1287475, MO1287476), OXF!, P! (P00410209), W!, fragment at CORD! = Capsicum rhomboideum (Dunal) Kuntze (Jørgensen and León-Yánez 1999)

Both syntypes cited in the original description are from the Andes of Ecuador. The specimen Spruce 5117 is deposited at K (K000201905!, K000201904!) and BM (BM000072741!) and apparently also at AWH. A note on sheet K000201904, states “Although Van Heurck quotes this number it does not agree with his description which answers to 5050 and is a Brachistus. N.E. Brown”. According to Knapp (2002: 395), Spruce 5117 belongs to Solanum nudum Dunal. The collection Spruce 5050 has been widely distributed in different herbaria, and is very complete. Two sheets of Spruce 5050 are held at Kew, where Spruce’s original set is deposited; K000201915 is the better sheet with more complete data in the label, and is here chosen as lectotype. Spruce has handwritten “baccae nigrae” on this sheet, and the dense pubescence, the rhomboidal to elliptic leaves, 5-toothed calyx, and campanulate corolla are clearly visible and match the protologue. The calyx with clearly observable teeth and anthers with longitudinal dehiscence are typical of the genus Capsicum and the combination of characters cited above fits under Capsicum rhomboideum. The mature fruits of this species are bright red more than black but often become purple or darker on dry specimens.

Capsicum campylopodium Sendtn., Fl. Bras. 10(6): 144. 1846. Type citation: [BRAZIL]. “In Serra d’Estrella, prov. Rio de Janeiro: Schott; in Brasilia australiore: Sellow”.- Lectotype (designated here): “Brasilia, Sellow 6”- P! (P00410022); isolectotypes: BM!, CORD!: fragment, K! (K000585891, right plant), F!: B photo n° 2865 + fragment.

Both syntypes were successfully located. Duplicates of Schott 5409 are housed at F!, GH!, and W!, and all of them are plants in flower. The Sellow collection has been distributed more widely; a photograph and a fragment (with fruit and seeds) of the Berlin specimen (“B 1543”) destroyed during World War II are held at F; the P sheet of Sellow includes flowering and fruiting material and has an original handwritten label assigning the number 6 to this collection. Of the two syntypes, the Sellow specimen at P is selected as lectotype since it is a complete one and it is possible to distinguish the most distinctive characters of Capsicum campylopodium (toothless calyx, geniculate pedicels at anthesis, and black seeds with spine-like projections) (Barboza and Bianchetti 2005).

Capsicum eximium Hunz., Darwiniana 9(2): 235. 1950. Type citation. “Argentina. Salta. Quebrada de San Antonio, Pampa Grande, 1600 m, Dpto. Guachipas, leg. Armando T. Hunziker nº 1907, 6 May 1942. Ejemplar fructificado. (ATH[unziker]).- Semillas del ejemplar nº 1907 cultivadas en el Jardín Botánico de la Facultad de Agronomía y Veterinaria de Buenos Aires, leg. Armando T. Hunziker 7346, 4 Mar 1943. Ejemplares floríferos (ATH[unziker])”.- Lectotype (designated here): “ARGENTINA: Semillas del ejemplar nº 1907 cultivadas en el Jardín Botánico de la Facultad de Agronomía y Veterinaria de Buenos Aires, A.T. Hunziker 7346, 4 Mar 1943” – CORD!; isolectotype: CORD!.

Hunziker cited two syntypes. The first collection (Hunziker 1907), is abundant consisting of 18 fruiting specimens held at CORD. The second one (Hunziker 7346) is here designated as lectotype; it originates from plants cultivated from seeds of the original collection and consists of two flowering branches on a single sheet. A duplicate of this latter collection is a small specimen also in flower. Fruiting specimens of Capsicum are not only difficult to distinguish from other species of Capsicum but sometimes also from some species of Lycianthes as both genera share the similar calyx features. The flowering collection is designated here as lectotype since the corolla is the best organ with which to characterize Capsicum eximium (Hunziker 1950).

Capsicum mirabile Sendtn., Fl. Bras. 10(6): 143. 1846. Type citation: [BRAZIL]. “In sylvis fere ubique per prov. Sebastianopolitana et Paulinam, Decembri florens: Martius, Karwinski; in irriguis lapidosis in Serra de S. Geraldo, prov. Minarum, Aprili florens: Martius”.- Lectotype (designated here): [BRAZIL]: “In sylvis fere ubique per prov. Sebastianopol. et Paulinam, Dec., Martius s.n.”- M!

**Figure 1.**
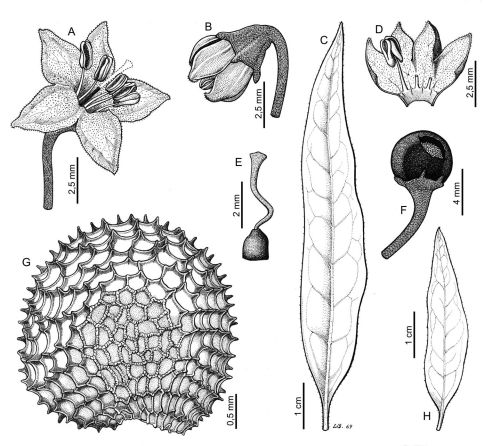
Capsicum mirabile. **A** flower **B** flower bud **C, H** leaf **D** open corolla **E** gynoecium **F** fruit **G** seed. Line drawing by L. Sánchez; voucher Martius s. n., lectotype.

The three syntypes were examined. Even though the second syntype, deposited at BR! ([Brazil] “Prope Rio de Janeiro, L.B. de Karwinski s.n., 1823”), matches the protologue, the Martius specimen at M is chosen here as the lectotype as it is more complete and better preserved. Both specimens show the characteristic features of Capsicum mirabile such as the narrowly elliptic and glabrous leaves (young leaves with scarce short hairs on the margins), geniculate pedicels, glabrous calyx with 5 long teeth, stellate corolla, and black seeds with spine-like projections (Fig. 1). The third syntype (the Martius collection from Serra de S. Geraldo, Minas Gerais [M!, M photo nº 6522 at F!]) is unusually pubescent for this species.

Capsicum villosum Sendtn. var. *muticum* Sendtn., Fl. Bras. 10(6): 145. 1846. Type citation: [BRAZIL]. “In Serra d’Estrella ejusdem prov. [prov. Sebastianopolitanae]: Schott; in Brasilia australiore: Sellow”.- Lectotype (designated here): [BRAZIL. Rio de Janeiro]: “Serra d’Estrella, Schott 5416”- W!

One of the two syntypes, the Sellow specimen at B (Sellow 79, in sched.), was destroyed and the only elements remaining of it are F neg. #2874 and an incomplete fragment at F! The other syntype (Schott 5416), here designated as the lectotype, is not a well-preserved specimen but the diagnostic characters of this variety (yellowish and long patent hairs on stem and pedicels, leaves densely pubescent in both surfaces, and angulate calyx with 5 short teeth), can be seen.

## A new name for Capsicum eximium var. tomentosum

### Capsicum eshbaughii

Barboza nom. nov.

urn:lsid:ipni.org:names:77109529-1

**Figure 2.**
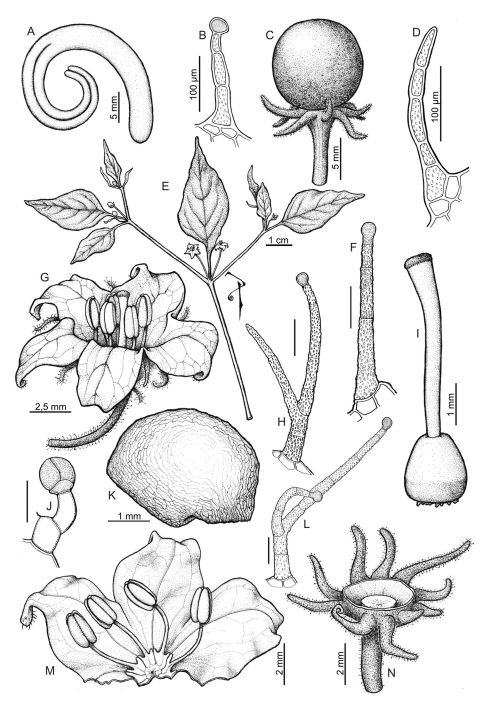
Capsicum eshbaughii. **A** embryo **B, H, F, J, L** glandular trichomes **C** fruit **D** non-glandular trichome **E** flowering branch **G** flower **I** gynoecium **K** seed **M** open corolla **N** fruiting calyx. Line drawing by P. Peralta; voucher A, C, K, N Nee 36164; B, D-J, L, M Eshbaugh 1943 b.

Capsicum eximium  var. *tomentosum* Eshbaugh & P.G.Sm., Baileya 18: 15. 1971, non Capsicum tomentosum Kuntze, 1891. - Holotype: BOLIVIA, Dpto. Santa Cruz, Prov. Florida, Mairana area, 1300 m, P.G.Smith Sa281 (holotype, IND!; isotypes: MU! (MU-153648, MU-153649). Fig. 2

#### General

**Figure 3.**
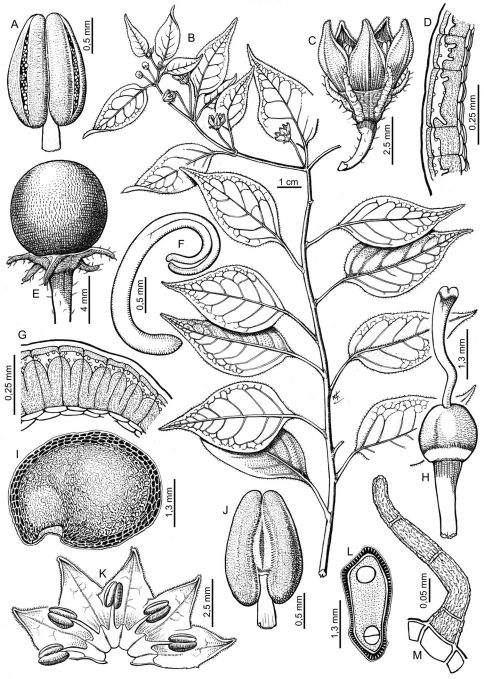
Capsicum eximium. **A, J** anthers in ventral and dorsal view respectively **B** flowering branch **C** flower **D, G** transverse section of the seed coat **E** fruit **F** embryo **H** gynoecium **I** seed **K** open corolla **L** seed in cross section **M** non-glandular trichome. Line drawing by N. Flury; voucher A, C-M: Hunziker 1907; B, Hunziker 7346.

Capsicum eximium var. *tomentosum* was described as an unusual glandular tomentose variety of Capsicum eximium Hunz. The presence of this kind of pubescence densely covering the vegetative organs, pedicels, and calyx, and of a 5–10 toothed-calyx (Fig. 2 N) in specimens from a restricted area in south-central Bolivia (Dpto. Santa Cruz and Cochabamba) makes them quite different from Capsicum eximium. In fact, Capsicum eximium has non- glandular pubescence (Fig. 3 M), a calyx with only 5 teeth (Fig. 3 C, E), and is distributed in a larger area (Bolivia: La Paz to Tarija and Argentina: Jujuy to Tucumán).

Glandular hairs are rare in Capsicum. The dense indumentum of Capsicum eshbaughii consists of different types of glandular trichomes, some of them observed only in this species. The hair variations are: long simple hairs with multicellular and verrucate stalks and unicellular stipitate (Fig. 2 F) or not stipitate (Fig. 2 B) heads; short hairs with bicellular smooth stalks and multicellular heads (Fig. 2 J); and bifurcate hairs with both branches ending in a unicellular head (Fig. 2 L) or one branch non-glandular and the other longer and glandular (Fig. 2 H).

Capsicum eshbaughii, together with Capsicum eximium and Capsicum cardenasii Heiser & P.G.Sm., is known as “ulupica” (Eshbaugh 1943 C); their very pungent fruits are very much appreciated as a spice or as pickles in the kitchens of Bolivia (Eshbaugh and Smith 1971).

This species is named in honor to Dr. W.H. Eshbaugh (Miami University) who first recognised the distinctness of this species and devoted part of his research to the taxonomy of chili peppers.

Specimens Examined. Bolivia. Cochabamba: José Carrasco Torrico, camino Cochabamba-Comarapa, Copachuncho, ca. 3000 m, 29 Mar 1987, D.Flores 89 (CORD, LPB). Santa Cruz: Florida, El Sauce, west of Samaipata, elev. 1730 m, 30 Mar 1987, W.H.Eshbaugh 1943 C & D (CORD); same locality, 1 Aug 1990, W.H.Eshbaugh 1943 a (CORD); 5 km (by air) SE of Mairana, on road to Samaipata, at “Quebrada Seca”, 18º 09’ S, 63º 56’ W, ca. 1550 m, 4 Feb 1988, M.Nee 36164 (CORD).

## Supplementary Material

XML Treatment for Capsicum eshbaughii

## References

[B1] Barboza GE Hunziker AT (1989) Estudios sobre Solanaceae XXIX. Sinopsis taxonómica de *Athenaea*. Boletín de la Sociedad Argentina de Botánica 26:91-105

[B2] Barboza GE Hunziker AT (1992) Estudios sobre Solanaceae XXXII. El género *Lycianthes* en la Argentina. Darwiniana 31 (1–4):17-34

[B3] Barboza GE Bianchetti LB (2005) Three new species of *Capsicum* (Solanaceae) and a key to the wild species from Brazil. Systematic Botany 30: 863–871 doi: 10.1600/036364405775097905

[B4] Barboza GE Agra MF Romero MV Scaldaferro MA Moscone EA (in press) New endemic species of *Capsicum* (Solanaceae) from the Brazilian caatinga: comparison with the re-circumscribed *C. parvifolium*. Systematic Botany.

[B5] Barceloux DG (2008) Pepper and capsaicin (*Capsicum* and *Piper* species). In: Barceloux DG (Ed) Medical Toxicology of Natural Substances: Foods, Fungi, Medicinal Herbs, Toxic Plants, and Venomous Animals. John Wiley & Sons Hoboken, New York, 71–76

[B6] Basu SK De AK (2003) *Capsicum*: historical and botanical perspectives. In: De AK (Ed) *Capsicum*: The genus *Capsicum*. Taylor & Francis, London & New York, 1–15

[B7] Bentham MG Hooker JD (1876) Genera Plantarum 2 (2). A. Black, W. Pamplin, Lovell Reeve & Co., Williams & Norgate, London, I-VIII, 533–1279

[B8] Bitter G (1919) Die Gattung *Lycianthes*. Abhandlungen herausgegeben vom Naturwissenschaftlichen Verein zu Bremen 24:292-520

[B9] Bitter G (1921) Aufteilung der Gattung *Bassovia* (im Dunalschen Sinne) zwischen *Solanum*, *Capsicum* und *Lycianthes*). Repertorium Specierum Novarum Regni Vegetabilis 17:328-335

[B10] Bosland PW Votava EJ (2000) Peppers: Vegetable and Spice Capsicums. Crops Production Science in Horticulture 12 CABI Publishing, Wallingford

[B11] Bosland PW Zewdie Y (2001) Diversity and characterization of capsaicinoids and their application to chemotaxonomy of *Capsicum*. In: van den Berg RG Barendse GWM van der Weerden GM Mariani C (Eds) Solanaceae V: Advances in Taxonomy and Utilization. Nijmegen University Press, Nijmegen, 179–185

[B12] Buckenhüskes HJ (2003) Current requirements on paprika powder for food industry. In: De AK (Ed) *Capsicum*: The genus *Capsicum*. Taylor & Francis, London & New York, 223–230

[B13] Chodat R (1902) Solanaceés. In: Chodate R (Ed) Plantae Hasslerianae soit énumération des plantes récoltées au Paraguay par le Dr ÉmilHasslerd’Aarau (Suisse) de1885–1895 et déterminées par le Prof. Dr R. Chodat avec l’aide de plusieurs collaborateurs. Bulletin de l’herbier Boissier ser. 2, 2 :745-747.; 811–815

[B14] Chodat R Hassler E (1903) Solanaceés. In: Chodat R Hassler E (Eds) Plantae Hasslerianae soit énumération des plantes récoltées au Paraguay par le Dr Émile Hassler d’Aarau (Suisse) et publiées par le Prof Dr. R. Chodat et le Dr. E. Hassler. Bulletin de l’herbier Boissier ser.2, 4 :77-89.

[B15] D’Arcy WG Eshbaugh WH (1973) The name of the common bird pepper. Phytologia 25: 350

[B16] D’Arcy WG Eshbaugh WH (1974) New World Peppers [*Capsicum*-Solanaceae] North of Colombia: a résumé. Baileya 19:93-105

[B17] Eshbaugh WH Smith PG (1971) A new variety of chili pepper, *Capsicum eximium* var. *tomentosum* (Solanaceae). Baileya 18:13-16

[B18] Dunal MF (1852) Solanaceae. In: Candolle AP de (Ed) Prodromus systematis naturalis regni vegetabilis, vol. 13 Sumptibus Victoris Masson, Paris, 1–690

[B19] Filippa EM Bernardello LM (1992) Fruto y semilla en *Athenaea*, *Aureliana* y *Capsicum*. Darwiniana 31:137-150

[B20] Heiser CH Jr. (1964) Los chiles y ajíes (*Capsicum*) de Costa Rica y Ecuador. Ciencia y Naturaleza 7:50-55

[B21] Heiser CH Jr. Pickersgill B (1969) Names for the cultivated *Capsicum* species (Solanaceae). Taxon 18:277-283

[B22] Heiser CH Jr. Pickersgill B (1975) Names for the bird pepper [*Capsicum*-Solanaceae]. Baileya 19:151-156

[B23] Heiser CH Jr. Smith PG (1958) New species of *Capsicum* from South America. Brittonia 10:194-201

[B24] Hunziker AT (1950) Estudios sobre Solanaceae. I. Sinopsis de las especies silvestres de *Capsicum* de Argentina y Paraguay. Darwiniana 9:225-247

[B25] Hunziker AT (1956) Synopsis of the genus *Capsicum*. Huitieme VIII Congrès International de Botanique. Paris. Compte Rendu des Séances, Rapport & Commentaries [Paris] 4 (2):73-74

[B26] Hunziker AT (1969) Estudios sobre Solanaceae. V. Contribución al conocimiento de *Capsicum* y géneros afines (*Witheringia*, *Acnistus*, *Athenaea*, etc.). Primera Parte. Kurtziana 5:101-179

[B27] Hunziker AT (1977) Estudios sobre Solanaceae. VIII. Novedades varias sobre tribus, géneros, secciones y especies de Sud América. Kurtziana 10:7-50

[B28] Hunziker AT (1982) Revisión sinóptica de *Acnistus*. Kurtziana 15:81-102

[B29] Hunziker AT (1984) Estudios sobre Solanaceae. XIX: Sinopsis de *Vassobia*. Kurtziana 17:91-128

[B30] Hunziker AT (1998) Estudios sobre Solanaceae. XLVI. Los ajíes silvestres de Argentina (*Capsicum*). Darwiniana 36:201-203

[B31] Hunziker AT (2001) Genera Solanacearum. The Genera of Solanaceae Illustrated, Arranged According to a New System. A. R.G. Gantner Verlag K.-G., Ruggell, 500pp.

[B32] Hunziker AT Barboza GE (1991) Estudios sobre Solanaceae XXX. Revisión de *Aureliana*. Darwiniana 30:95-113

[B33] Jørgensen PM León-Yánez S (1999) Catalogue of the Vascular Plants of Ecuador. Monographs in Systematic Botany from the Missouri Botanical Garden 75: I–VIII, 1–1181

[B34] Kuntze O (1891) Revisio Generum Plantarum, part II. Commissionen. A. Felix, Leipzig, 377–1011

[B35] Knapp S (2002) *Solanum* section *Geminata* (Solanaceae). Flora Neotropica Monograph 84:1-404

[B36] Linné C (1753) Species Plantarum, vol. 1 Salvias, Holmia, 560pp.

[B37] Manirakiza P Covaci A Schepens P (2003) Pungency principles in *Capsicum* - Analytical determinations and toxicology. In De AK (Ed) *Capsicum*: The genus *Capsicum*. Taylor & Francis, London & New York, 71–86

[B38] McNeill J Barrie FR Burdet HM Demoulin V Hawksworth DL Marhold K Nicolson DH Prado J Silva PC Skog JE Wiersema JH Turland NJEds (2006) International Code of Botanical Nomenclature (Vienna Code): Adopted by the Seventeenth International Botanical Congress Vienna, Austria, July 2005. Regnum Vegetabile 146 Gantner, Ruggell

[B39] McMullen CK (1999) Flowering Plants of the Galápagos. Cornell University Press, Ithaca and London, 370pp.

[B40] Miers J (1849) Contributions to the botany of South America. Annals and Magazine of Natural History Ser. II, 3 (16):261-269

[B41] Morton CV (1944) Taxonomic studies of tropical American plants. Contributions from the United States National Herbarium 29 (1):1-86

[B42] Moscone EA Scaldaferro MA Gabriele M Cecchini NM Sánchez García Y Daviña JR Ducasse DA Barboza GE Ehrendorfer F (2007) The evolution in chili peppers (*Capsicum*-Solanaceae), a cytogenetic perspective. Acta Horticulturae (ISHS) 745: 137–170 http://www.actahort.org/books/745/745_5.htm

[B43] Perry L Dickau R Zarrillo S Holst I Pearsall DM Piperno DR Berman MJ Cooke RG Rademaker K Ranere AJ Raymond JS Sandweiss DH Scaramelli F Tarble K Zeidler JA (2007) Starch fossils and the domestication and dispersal of chili peppers (*Capsicum* spp.) in the Americas. Science 315: 986–988 doi: 10.1126/science.1136914 17303753 10.1126/science.1136914

[B44] Pruthi JS (2003) Chemistry and quality control of *Capsicum* and *Capsicum* products. In: De AK (Ed) *Capsicum*: The genus *Capsicum*. Taylor & Francis, London & New York, 25–70

[B45] Ravishankar GA Suresh B Giridhar P Ramachandra Rao S Sudhakar Johnson T (2003) Biotechnological studies on *Capsicum* for metabolite production and plant improvement. In: De AK (Ed) *Capsicum*: The genus *Capsicum*. Taylor & Francis, London & New York, 96–128

[B46] Reifschneider FJB (2000)Capsicum. Pimentas e pimentões no Brasil. Embrapa Comunicação para Transferência de Tecnologia, Brasilia, 113pp.

[B47] Ruiz H Pavón JA (1799) Flora Peruviana et Chilensis 2, Gabrielis de Sancha, Madrid, 1–76

[B48] Rusby HH (1926) Additions to the genus *Lycianthes* Dunal. Bulletin of the Torrey Botanical Club 53:209-213

[B49] Shinners LH (1956) Technical names for the cultivated *Capsicum* species (Solanaceae). Baileya 4:81-83

[B50] Thampi PSS (2003) A glimpse of the world trade in *Capsicum*. In: De AK (Ed) *Capsicum*: The genus *Capsicum*. Taylor & Francis, London & New York, 16–24

[B51] Tournefort JP (1719) Institutiones rei Herbariae 1 É Typographia regia, Paris, 695pp.

[B52] Walsh BM Hoot SB (2001) Phylogenetic relationships of *Capsicum* (Solanaceae) using DNA sequences from two noncoding regions: the chloroplast atpB-rbcL spacer region and nuclear waxy introns. International Journal of Plant Science 162:1409-1418

[B53] Wettstein R von (1891) Solanaceae. In: Engler A Prantl K (Eds) Die Natürlichen Pflanzenfamilien 4, 3b Verlag von Wilhelm Engelmann, Leipzig, 4–38

[B54] Yamamoto S Nawata E (2009) Use of *Capsicum frutescens* L. by the indigenous peoples of Taiwan and the Batanes Islands. Economic Botany 63: 43–59 doi: 10.1007/s12231-008-9052-5

